# Obstructive Sleep Apnea, Hypercoagulability, and the Blood–Brain Barrier

**DOI:** 10.3390/jcm10143099

**Published:** 2021-07-14

**Authors:** Cindy Zolotoff, Laurent Bertoletti, David Gozal, Valentine Mismetti, Pascale Flandrin, Frédéric Roche, Nathalie Perek

**Affiliations:** 1 U1059, Sainbiose, Dysfonction Vasculaire et Hémostase, Université de Lyon, Université Jean Monnet Saint-Étienne, F-42270 Saint-Priest-en-Jarez, France; laurent.bertoletti@gmail.com (L.B.); frederic.roche@univ-st-etienne.fr (F.R.); nathalie.perek@univ-st-etienne.fr (N.P.); 2 Service de Médecine Vasculaire et Thérapeutique, CHU Saint-Étienne, F-42270 Saint-Priest-en-Jarez, France; 3 Department of Child Health and the Child Health Research Institute, MU Women’s and Children’s Hospital, University of Missouri, Columbia, MO 65201, USA; gozald@health.missouri.edu; 4 Service de Pneumologie et d’Oncologie Thoracique, CHU Saint-Étienne, F-42270 Saint-Priest-en-Jarez, France; valentine.mismetti@gmail.com; 5 Laboratoire d’Hématologie, Hôpital Nord, CHU Saint-Étienne, F-42270 Saint-Priest-en-Jarez, France; pascale.flandrin-gresta@chu-st-etienne.fr; 6 Service de Physiologie Clinique et de l’Exercice, Centre VISAS, CHU Saint Etienne, F-42270 Saint-Priest-en-Jarez, France

**Keywords:** obstructive sleep apnea (OSA), coagulation, blood–brain barrier, procoagulant states, neurodegenerative diseases

## Abstract

Obstructive sleep apnea (OSA) is characterized by repeated episodes of intermittent hypoxia (IH) and is recognized as an independent risk factor for vascular diseases that are mediated by a multitude of mechanistic pathophysiological cascades including procoagulant factors. The pro-coagulant state contributes to the development of blood clots and to the increase in the permeability of the blood–brain barrier (BBB). Such alteration of BBB may alter brain function and increase the risk of neurodegenerative diseases. We aim to provide a narrative review of the relationship between the hypercoagulable state, observed in OSA and characterized by increased coagulation factor activity, as well as platelet activation, and the underlying neural dysfunction, as related to disruption of the BBB. We aim to provide a critical overview of the existing evidence about the effect of OSA on the coagulation balance (characterized by increased coagulation factor activity and platelet activation) as on the BBB. Then, we will present the emerging data on the effect of BBB disruption on the risk of underlying neural dysfunction. Finally, we will discuss the potential of OSA therapy on the coagulation balance and the improvement of BBB.

## 1. Introduction

Obstructive sleep apnea (OSA) is a highly prevalent medical disease that imposes large arrays with adverse socioeconomic impacts, particularly in developed countries [1]. It affects, albeit differently, both sexes and all age groups in the population. Middle-aged patients with OSA who are also evaluated at high risk for cardiovascular diseases are a particularly important group to diagnose and treat in light of their uniquely elevated risk of both cardiovascular and cerebrovascular morbidity and mortality [2]. In this high-risk group, there also appears to be a significant and independent association between OSA and cognitive complaints, as well as neurodegenerative diseases [3,4].

According to the World Health Organization, about 18 million people will die each year due to cardiovascular diseases (CVD), accounting for 31% of all deaths around the world [5]. Thrombosis, due to an abnormally activated coagulation cascade, is an important part of their pathophysiological mechanisms [6,7]. In parallel with the prominent role played by CVD, an overview of the studies performed to date clearly shows a significant relationship between untreated, severe OSA and all-cause mortality [8].

The changes in coagulation balance observed in OSA could have potential consequences on the cardiovascular system but may also directly affect the brain [9]. Increased activity of clotting factors and platelets may also negatively impact the blood–brain barrier (BBB) by an increase in its permeability [10,11]. As a result, compounds that are potentially harmful to the brain and usually blocked by the BBB may contribute to the development or worsening of neurodegenerative diseases [12].

Based on the aforementioned considerations, this paper aims to provide a comprehensive narrative review of the potential links between OSA, abnormally activated coagulation, and platelets, as their potential consequences on the brain, because of BBB disruption.

## 2. OSA: Definition, Clinical Consequences, and Treatments

Among the vast spectrum of sleep-related breathing disorders (SRBD), OSA is the most frequent condition. A recent study has estimated that nearly a billion people worldwide suffer from OSA [13]. OSA is characterized by recurrent obstruction of the upper airway during sleep leading to either apnea (cessation of airflow) or hypopnea (substantial decrease of the airflow with consequent oxyhemoglobin desaturation and/or electroencephalographic arousal). These apneas/hypopneas lead to sleep fragmentation and its downstream daytime functional consequences. The severity of the disease is reported using the apnea and hypopnea index (AHI), i.e., the number of respiratory events per hour of sleep.

The dominant nocturnal symptom of OSA is snoring with a prevalence of 75–90% in OSA patients [14]. Furthermore, nocturnal symptoms can also include apnea observed by bed partners, paroxysmal dyspnoea, frequent awakenings, excessive perspiration, restlessness, and nightmares. Daytime symptoms characteristically consist of excessive sleepiness and unexplained fatigue. Moreover, patients report morning headaches (12–18%), dryness of the oral mucous membranes, impairment of cognitive functions [15], recurrent problems with memory and concentration, mood deterioration, and a tendency towards depressive symptoms [16].

Repeated episodes of hypoxia–reoxygenation in these patients promote oxidative stress, which is the result of excessive production of reactive oxygen species (ROS). Moreover, oxidative stress is involved in the regulation of cellular transcription through the activation of transcription factors such as HIF-1 (hypoxia-inducible factor-1), which is activated by hypoxia and is responsible for the activation of several genes, including vascular endothelial growth factor (VEGF) [17]. Indeed, high serum levels of HIF-1α protein are compatible with the diagnosis of OSA, whereas low levels may exclude severe OSA with high probability [18]. HIF-1 α can also stimulate the transcription factor NF-κB involved in the regulation of inflammatory responses [19]. Stimulation of systemic inflammatory pathways favors the induction of endothelial dysfunction, increased blood coagulability, insulin resistance, activation of monocytes and macrophages fostering the development of atherosclerosis, as well as stimulation of the renin–angiotensin–aldosterone system [20].

Overnight polysomnography remains the “gold standard” for the diagnosis of OSA, even if ambulatory polysomnography and particularly respiratory polygraphic recordings have superseded in-lab studies in general clinical practice [21].

Current therapies for OSA, such as continuous positive airway pressure (CPAP) and oral appliances, have relatively poor long-term adherence as well as variable efficacy. The risk:benefit ratio of such interventions for cardiovascular event prevention (primary or secondary) is still under debate, with randomized clinical studies yielding negative results [22,23]. However, in stroke secondary prevention, the efficacy of CPAP is well recognized when the mean night CPAP treatment duration is 4 > h/night [24]. When patients have mild to moderate sleep apnea and CPAP is frequently poorly tolerated, an endo-buccal device may be an alternative treatment. This is a so-called advancement or retention mandibular orthosis that holds the tongue and lower jaw forward, thus facilitating the retropharyngeal airflow passage [25]. This treatment is less restrictive, but also less effective, than CPAP for severe forms of the syndrome. Other treatments exist, such as surgery, to increase the cross-sectional area of the upper airways by removing excess tissue or advancing the upper and lower jaws [26]. Moreover, bilevel positive airway pressure (BiPAP) is a better option for some people. This noninvasive ventilation treatment delivers an adaptative inspiratory pressure and an expiratory pressure level [27]. The search for novel therapeutic approaches for OSA, including pharmacological agents, has been actively pursued over the past years, further highlighting the importance of cell or animal models of OSA, their applicability, and limitations [28].

## 3. OSA and the “Hypercoagulable State”

OSA increases the risk of cardiovascular diseases via different pathways, including oxidative stress, inflammation, and coagulation abnormalities. Hypoxia can directly activate clotting [29], especially in IH, such as occurs in chronic obstructive pulmonary disease exacerbations [30,31,32]. Furthermore, in some patients with heart failure, the vascular endothelium seems to have procoagulant properties [33]. In patients with OSA, several factors are altered, including hematocrit [34] and blood viscosity [35,36,37], but these two are not linked to BBB damage. Thus, in this section, we will delineate the main factors affected during OSA that are involved in the alteration of the BBB.

### 3.1. Factors Contributing to a Procoagulant State in OSA

#### 3.1.1. Clotting Factors

The thrombin–antithrombin (TAT) complex is formed in response to a high level of thrombin suggesting increased coagulation. Thus, the TAT complex is a good indicator for measuring the level of thrombin in the blood. Several studies [38,39], including one randomized study with 220 patients who suffered from OSA [38], have demonstrated that levels of TAT were higher in patients with OSA.

Other important clotting factors are factors VII (FVIIa) and XII (FXIIa). They are essential components of the coagulation cascade. Interestingly, both FVIIa [40] and FXIIa [41,42] have been associated with an increase in arterial disease, and elevated levels of these factors have been reported in randomized trials in patients with OSA [38,43] and might account for high cardiovascular morbidity of OSA.

Fibrinogen is also a major coagulation protein; once converted into fibrin, it allows for clot formation and influences platelet aggregation. Thus, fibrinogen levels appear to be an important risk factor for cardiovascular disease [44,45]. Various studies have found that fibrinogen is increased in patients with OSA [35,46,47,48,49,50,51] and circulating fibrinogen levels are linearly correlated with AHI in different case–control studies [52,53,54,55].

#### 3.1.2. Platelets

Platelets are a blood component whose major function is to agglutinate during a blood vessel injury, triggering the formation of a blood clot to stop the bleeding. After the adhesion of platelets to the endothelium, platelet activation is observed. Platelets contain many cell adhesion and inflammatory factors that can be released when they are activated, including soluble CD40 ligand (sCD40L) and P-selectin. In different studies, subjects with OSA have found platelet activity to be increased in these patients [56,57,58] and correlated with an increase in sCD40L [59,60,61] and P-selectin [62,63,64,65] levels. A few minutes after activation, platelet aggregation is observed. During this stage, several factors are released, including platelet-activating factor (PAF) and adenosine diphosphate (ADP), both of which contribute to the maintenance of platelet aggregation. In patients with OSA, platelet aggregation is increased in nonrandomized studies [46,57,66,67,68], particularly induced by an increase of ADP [67,69]. In an in vitro model study, hypoxia–reoxygenation (H/R) induced increases in PAF levels [70]. In a mouse model of OSA, transgenic mice that were deficient in the cell surface receptor for PAF (PAFR-/-) showed attenuated elevations of inflammatory signaling [71]. It would therefore appear that a coordinated activity of all these elements contributes to the development and propagation of thrombotic phenomena in the context of OSA [72]. Given their involvement in inflammation, several data confirm that blood platelets in OSA patients are a therapeutic target to reduce the risk of cardiovascular disease [17].

#### 3.1.3. Von Willebrand Factor

Von Willebrand factor (VWF) is an adhesion molecule that circulates in plasma and has a central role in primary hemostasis. It mediates platelet adhesion during vascular injury and allows the transport and stabilization of circulating factor VIII [73]. Although some studies show no differences between healthy subjects and OSA patients [38,74], more recent studies have shown a significant increase in VWF in sleep apnea [49,75,76,77].

Notwithstanding, it is important to emphasize that there are many confounding factors that cooccur among OSA patients, such as hypertension, diabetes, smoking, and obesity, all of which can also directly affect the blood clotting system. Thus, based on the extant evidence, it is possible that OSA may accelerate this process.

### 3.2. Effects of CPAP on Coagulation Balance

The effect of CPAP on the hypercoagulable state associated with OSA has been evaluated in numerous biological studies. CPAP treatment was accompanied by a decrease in FVII [43], fibrinogen [43,46], and VWF (57). However, no beneficial effects were observed regarding factor XII [38] and thrombin [38]. Concerning platelet activation, a significant decrease in sCD40L [59,61] and P-selectin [63] was observed. Platelet aggregation also decreased in several studies [46,66,67] (see Table 1). The clinical impact of such improvement is still poorly evaluated. Moreover, as with any treatment, the beneficial effects of CPAP are intimately related and dependent on adherence, the impact of possible comorbidities, as well as on the underlying severity of OSA. Moreover, they could come in part from the chronic restoration of a normal nocturnal respiratory function.

## 4. Impact of OSA on BBB

### 4.1. Blood–Brain Barrier

A recent review and other studies have shown that the blood–brain barrier (BBB) could be altered by several mechanisms that are present during OSA [78,79,80]. The BBB secludes the brain from the undesired transfer of substances that may be in the bloodstream while allowing the passage of nutrients that are essential for brain function. This protective barrier is constituted by several elements that are essential for maintaining the tightness between the capillary and the cerebral space, as well as regulate the transport between these two compartments. Among these elements, endothelial cells, astrocytes, pericytes, and neurons account for the most important [78]. Endothelial cells are held together by tight junctions (TJs), which create a paracellular barrier of high resistance to limit permeability. The transmembrane proteins that make up the TJs (zonula occludens (ZO)-1, claudin-5, etc.) limit the paracellular transfer of molecules. TJs also interact with adherent basal junctions (e.g., vascular endothelial cadherin) to strengthen the interactions between endothelial cells [81].

Many polarized metabolites enter the brain by facilitated diffusion. However, the BBB also has an exceptional ability to protect the brain from potentially toxic xenobiotics and metabolites through efflux transporters. For this reason, endothelial cells also express transport proteins such as ATP binding cassette (ABC) transporters [82]. These proteins are mainly located on the luminal membrane of the brain microvessels and they recognize a wide range of different substrates, allowing them to be transported from the central nervous system (CNS) to the bloodstream. They include P-glycoprotein, breast cancer resistance protein, and multidrug resistance proteins [82]. All those microstructural cell elements are essential to ensure the integrity of the BBB.

Dysfunction of the BBB will lead to an increase in membrane permeability and the potential entry of cells and molecules into CNS, resulting in neuronal dysfunction and degeneration [83].

### 4.2. OSA: Adverse Effects on BBB Function

Alterations in BBB in the context of OSA are thought to promote the emergence of cognitive impairments and may be associated with several neurodegenerative diseases [78].

Oxidative stress, as induced by OSA, is characterized by increases in ROS generation and propagation and causing reducing nitric oxide production [84]. In addition to oxidative stress, another pathway through which IH can alter cell function is via changes in molecular oxygen sensors. Indeed, HIF-1α transcription is stabilized during the early stages of IH [85], and its binging to the promoter regions of regulated genes enhances the transcription of such genes, which are involved in various biological processes such as inflammation or cancer [78]. Furthermore, chronic inflammation may occur in the BBB in response to IH and act as another mechanism leading to cognitive impairments. IH results in the activation of the transcription factor NFκB [86] and other transcription factors underlying immune responses, resulting in increased levels of proinflammatory cytokines among OSA patients [87,88].

The consequences of the aforementioned process on the BBB are multiple and are primarily characterized by changes in the permeability of the BBB capillary network, as well as modifications in ABC transporters [78]. There is also leakage through the paracellular pathway and therefore through TJs [78]. Studies in mice exposed to IH have shown increases in parenchymal water in the brain, as well as alterations in aquaporin expression, leading to increased permeability of the BBB [89]. Changes in the permeability of the BBB were also inferred from studies in adult patients with OSA [90].

Very recent studies have shown that endothelial cells secrete exosomes and that endothelial cells can also be targeted by exosomes derived from different cell types. Exosomes are a class of very small extracellular vesicles, with a diameter of 30–100 nanometers [91]. It is now recognized that stress conditions can disrupt the endothelial TJs of the brain and affect cognition via exosome-related biological activities [92]. Recently, Khalyfa et al. [93] demonstrated that extracellular vesicles (EVs) (including exosomes) are increased in the plasma of children with OSA. These EVs disrupt the integrity of the BBB by imposing adverse effects on the integrity of the monolayer of the endothelial barrier while also disrupting the TJ structure.

### 4.3. Hypercoagulability and Possible Effects on BBB

**Figure 1:**
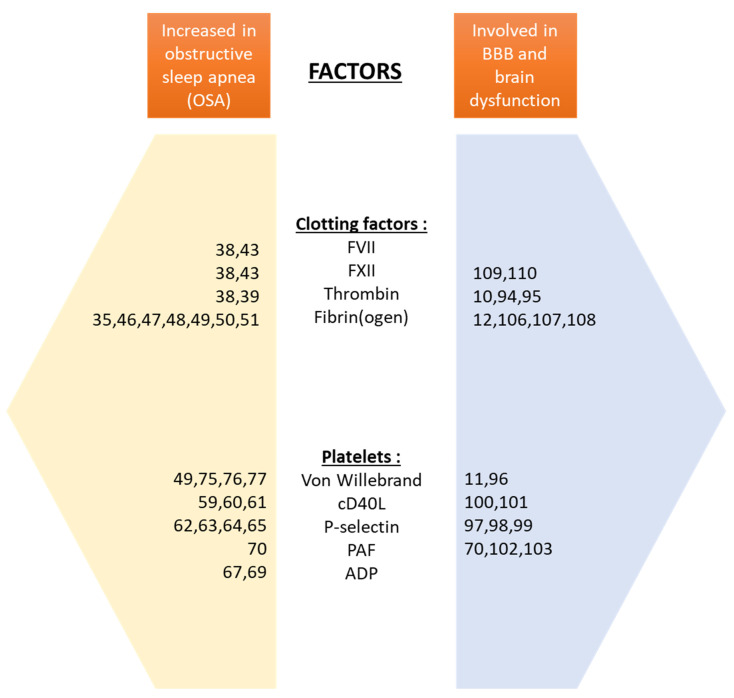
Summary of the major studies investigating the factors increased during obstructive sleep apnea (OSA) and factors involved in blood–brain barrier (BBB)–brain dysfunction. ADP: adenosine diphosphate, PAF: platelet-activating factor, cD40L: cD40 ligand.

Currently, we are unaware of any studies establishing a direct link between OSA, a prothrombotic state, and alterations in BBB function and structure. However, several studies involving conditions unrelated to OSA have demonstrated an alteration of the BBB when the concentrations of procoagulant factors are increased (see Figure 1).

In a study evaluating biomarkers leading to BBB dysfunction in cognitively impaired patients, serum thrombin levels were found to be abnormally elevated. The direct effects of this molecule on the integrity of the BBB were tested in vitro using microvascular endothelial cells. The permeability of the BBB was altered, demonstrating the role of thrombin and its ability to disrupt BBB function [10]. In adults rats, thrombin injection resulted in disruption of brain microvascular endothelial cells and the BBB, as evidenced by increased permeability of the BBB and increased brain water content [94]. Another study showed that after the injection of thrombin into the basal ganglia in rats, there was a significant increase in the expression of matrix metalloproteinases, which are involved in the disruption of the BBB. This perturbation of the BBB is then associated with the formation of CNS edema in this experimental model [95].

VWF is also known to alter the BBB. In a mouse model subjected to hypoxia–reoxygenation (H/R) episodes, VWF deficiency showed an increased expression of claudin-5 in endothelial cells. VWF deficiency thus confers partial preservation of the integrity of the BBB after H/R [96]. Another study identified a critical role of VWF in brain inflammation associated with altered BBB after intracerebral hemorrhage. On the other hand, antibodies blocking VWF allowed limiting the lesions observed after hemorrhage [11].

Over-activated platelets, including the multiple factors they secrete, also play a significant role in BBB damage. Soluble P-selectin (sP-selectin) is a biomarker of platelet activation and is considered a risk factor for vascular disease. One study used a mouse model in which the endogenous P-selectin gene was replaced by a mutant that produces abnormally high plasma levels of sP-selectin. These mice then showed a higher permeability of the BBB that was associated with a higher risk of brain infarction and with the development of atherosclerotic lesions [97]. The increase in sP-selectin in these mice was also associated with shorter plasma clotting times and increased fibrin deposition on platelet thrombi, reflecting a procoagulant phenotype. Moreover, increased fibrin deposition in the brain in a mouse model of Alzheimer’s disease (AD) has been found to be associated with increased BBB permeability [98]. Another study confirmed that P-selectin expression contributed to early BBB dysfunction after stroke by using multimodality imaging approaches consisting of molecular magnetic resonance imaging and immunohistochemistry [99].

A study looking at the potential effects of sCD40L on the BBB used an in vitro model of human brain microvascular endothelial cells. The permeability of the cellular monolayer in this model was increased by the administration of sCD40L. Thus, sCD40L induces more severe inflammation of the CNS by disrupting the BBB [100]. Another study revealed that sCD40L levels were elevated in the circulation of HIV-infected and cognitively impaired individuals, compared to controls. Using microscopy and quantitative analyses in CD40L-deficient wild-type mice, it was found that the HIV trans-activator of transcription (Tat) can induce increased permeability of the BBB in a CD40L-dependent manner. The increased BBB permeability was found to be the consequence of abnormal platelet activation induced by Tat since pretreatment platelet depletion reversed the effects on BBB permeability [101].

In an in vitro study of the BBB consisting of rat brain microvessel-derived endothelial cells (RBMEC), an increase in PAF was demonstrated after H/R episodes. This increase was associated with endothelial cell damage. In addition, pretreatment with a PAF inhibitor suppressed the deleterious effects of PAF, leading to a protective effect on the BBB [70]. Moreover, immunohistochemical studies on RBMEC revealed that PAF reduced the immunostaining of ZO-1 (tight-junction-associated protein), increased F-actin fibers, creating leakage through the paracellular pathway [102]. In vivo studies have also indicated that PAF increases the permeability of BBB, as evaluated by sodium fluorescein and Evans blue methods [102]. Finally, intravenous infusion of PAF induced a transient opening of the BBB in rats, as reflected by an increased leakage of Evans blue and slight brain edema formation. PAF may also induce a transient and reversible opening of the BBB by a sharp decrease in regional cerebral blood flow [103].

Taken together, these studies show that a variety of procoagulant factors alter the permeability of the BBB via activation of multiple mechanisms, all of which may be relevant for inflammatory disorders of the CNS as well as deleterious to cognitive functioning.

### 4.4. BBB Disruption, Entry of Fibrinogen, and Neurodegenerative Diseases

**Figure 2:**
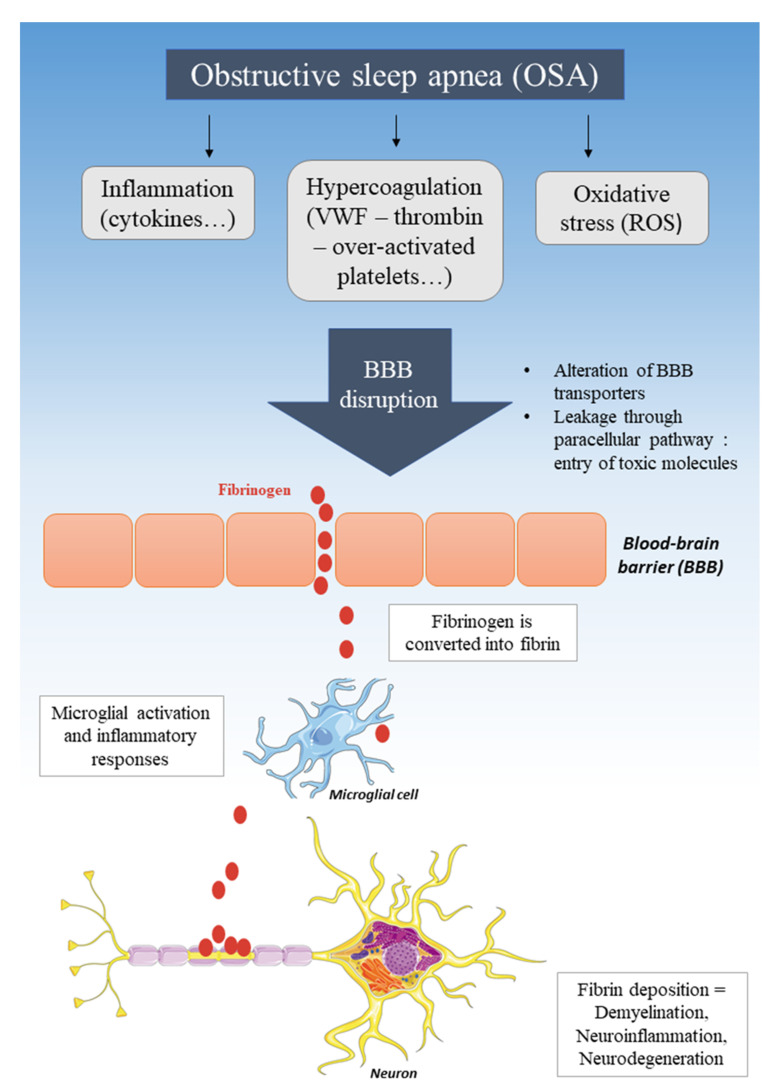
Pathogenic mechanism of obstructive sleep apnea (OSA) leading to blood–brain barrier (BBB) disruption and neurodegeneration. ROS: reactive oxygen species, VWF: von Willebrand factor.

Numerous studies have shown significant associations between neurodegenerative diseases and OSA. Indeed, in patients suffering from multiple sclerosis (MS) [104] or Alzheimer’s [105], the risk of OSA seems to be high. Numerous studies have suggested that after the BBB is disrupted, fibrinogen comes into contact with the white matter. Then, under those conditions, fibrinogen is converted to fibrin plaques and is then deposited on the CNS tissue. Fibrin induces neuroinflammation by activating microglia and by promoting the recruitment, migration, and activation of peripheral inflammatory macrophages [106]. The appearance of these fibrin plaques and the inflammatory cells that infiltrate the brain after a breach of the BBB is then associated with demyelination and neuronal dysfunction observed in these neurodegenerative diseases [12,107,108] (see Figure 2).

Several studies showed that this conversion of fibrinogen is possible through factor XII. In a mouse model of AD, FXII plays a key role in inflammatory-mediated neuronal damage and cognitive impairments [109]. In addition, similar to fibrinogen depletion, FXII depletion decreases neuroinflammatory responses and the resulting brain pathology [110].

Moreover, recent studies have indicated that REM-sleep-related apnea/hypopnea without atonia is very common in OSA and may represent an increased risk for neurodegenerative disease [4].

## 5. Potential Therapies

OSA has been identified as an independent risk factor for a large number of diseases. Among these, acute coronary syndrome (ACS) [111], atrial fibrillation (AF) [112,113], stroke [114], and venous thromboembolism (VTE) [115,116] are prominently represented.

As previously discussed, OSA is associated with an increased level of platelet volume indices and with platelet reactivity linked to the AHI index [111,117]. These findings may have direct involvement in the elevated prevalence of ischemic complications among ACS patients with OSA. In ACS patients with OSA, dual aspirin and clopidogrel therapy may be effective in reducing thromboembolic complications. However, in a clinical trial, there was a reduction in the antiplatelet effects induced by clopidogrel and a greater occurrence of high residual platelet reactivity during treatment in apneic patients [111]. These findings may explain why some ACS patients with OSA have a worse clinical prognosis than those in whom OSA is not present, suggesting the need to differentiate ACS patients with and without OSA for improved precision therapeutics [111]. In addition, patients with OSA required a significantly higher dose of warfarin than their non-OSA counterparts to limit the risk of recurrent pulmonary embolism [118].

In addition, in preclinical studies, dabigatran, apixaban, and rivaroxaban demonstrated a decrease in the opening of the BBB in cases of vascular dysfunction such as bleeding conditions [119,120,121,122,123]. Based on these results, several authors posited that such treatments may promote the preservation of cognitive function. AD and OSA show similarities in vascular dysfunction that contribute to dementia and cognitive impairments, including disruption of the BBB [124]. Several preclinical studies have demonstrated that the use of dabigatran, a direct thrombin inhibitor may be beneficial in AD, and could also be of value as adjuvant therapy in OSA patients manifesting cognitive dysfunction. In AD mice, long-term use of dabigatran has been shown to preserve memory and brain perfusion with an improvement in the integrity of the BBB [125]. These positive effects are associated with decreased levels of fibrin, amyloid deposition, and neuroinflammatory activity in the brains of these mice [125].

## 6. Conclusions

**Figure 3:**
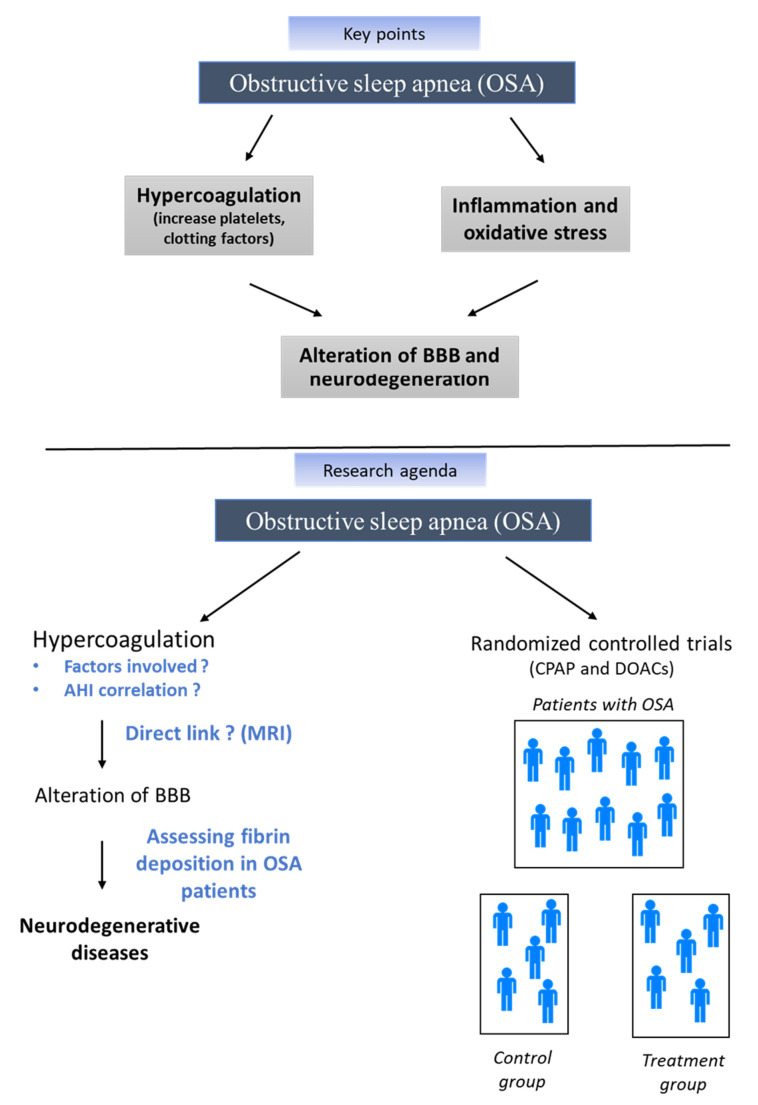
Key points and future directions, AHI: apnea–hypopnea index, BBB: blood–brain barrier, CPAP: continuous positive airway pressure, MRI: magnetic resonance imaging, OSA: obstructive sleep apnea.

We have summarized the evidence showing that OSA may promote a prothrombotic state that can lead to BBB damage. Oxidative stress, inflammation, and hypercoagulation pathways generated by OSA are not inconsequential and can all lead to an alteration of the BBB. We have further highlighted a disruption of the BBB in the context of increased concentrations of procoagulant factors, which then facilitate the entry of compounds potentially damaging to the brain, such as fibrinogen, deposition of fibrin on neurons, ultimately promoting accelerated neurodegeneration, as found in several diseases such as AD or MS. OSA is an eminently complex chronic disease associated with different phenotypes and therefore is involved in subsequent complications and increased risk of neurodegenerative diseases, which vary greatly from one patient to another. Then, it is important to note that a multiplicity of factors interact in OSA patients to create hypercoagulable and proinflammatory states that could influence BBB disruption and thus increase the risk of such neurodegenerative diseases. Thus, both individual factors and differences (demographic, genetic, and other early life risk markers) [126], modifiable risk factors (mood, sleep, diet, and lifestyle), and other vascular comorbidities that have been separately associated with downstream risk of hypercoagulable states should be considered in future studies. This detailed individual risk assessment will undoubtedly be a key element in the predictive medicine of tomorrow in OSA [127]. This will undoubtedly help the clinician to choose the most suitable treatment for OSA but also complementary drug therapies. For this reason, further studies need to be conducted to investigate the direct impact of OSA associated with hypercoagulation on brain tissue and to evaluate the impact of CPAP on the occurrence of thrombotic and cerebral pathologies (see Figure 3).

## Figures and Tables

**Table 1:** Hemorheological and coagulation findings in obstructive sleep apnea (OSA) and the effect of continuous positive airway pressure (CPAP).

Increased Factors in Osa	Authors	Type of Study—Subjects	CPAP Used? (>4 hours/night) Effects
FVII and FXII	Chin, 1998 [43]	Nonrandomized, controlled trial—15 males with OSA	Yes, decreased FVII levels after six months of CPAP
	Robinson, 2004 [38]	Randomized controlled trial—220 patients with OSA	Yes, no effects on FVII or FVIIa levels after one month CPAP
Thrombin	Robinson, 2004 [38]	Randomized controlled trial—220 patients with OSA	Yes, no effects on thrombin levels after one month
	Von Kanel, 2005 [39]	Uncontrolled intervention study—32 patients with OSA	No
Fibrinogen	Bouloukaki, 2017 [55]	Cross-sectional study—858 patients with OSA	No
	Chin, 1998 [43]	Uncontrolled intervention study—11 patients with OSA	Yes, decreased in fibrinogen levels after one night
	Comondore, 2009 [48]	Randomized crossover trial—13 patients with OSA	Yes, no effects in fibrinogen after 4 weeks CPAP
	Hizli, 2020 [50]	Randomized crossover trial—126 patients with OSA	No
	Mehra, 2010 [51]	Cross-sectional study—537 patients with OSA	No
	Nobili, 2000 [47]	Case-control study—12 patients with OSA	No
	Reinhart, 2002 [35]	Case-control study—13 patients with OSA	Yes, no effects in fibrinogen level after one night
	Shamsuzzaman, 2014 [54]	Case-control study—36 men with OSA	No
	Steiner, 2005 [52]	Case-control study—63 patients with OSA	No
	Von Kanel, 2016 [49]	Longitudinal study—329 patients with OSA	No
	Wessendorf, 2000 [53]	Case-control study—69 patients with OSA	No
	Zhang, 2003 [46]	Nonrandomized—41 patients with OSA	Yes, decreased in fibrinogen levels, after 30 days CPAP
Platelet activation	Bokinsky, 1995 [57]	Non-randomized study—6 patients with OSA	Yes, no effects
	Geiser, 2002 [56]	Case-control study—12 patients with OSA	No
sCD40L	Akinnusi, 2009 [59]	Non-randomized study—12 patients with OSA	Yes, decreased in sCD40L after 8 weeks
	Kobayashi, 2006 [61]	Case-control study—35 patients with OSA	Yes, decreased in sCD40L after 1 night
	Kosacka, 2015 [60]	Case-control study—79 OSA patients	No
P-selectin	Cofta, 2013 [62]	Group comparaison study—80 patients with OSA	No
	Horváth, 2020 [64]	Case-control study—51 patients with OSA	No
	Shimizu, 2002 [63]	Non-randomized study—94 patients with OSA	Yes, decreased in P-selectin after 1 month
	Winiarska, 2020 [65]	Group comparaison study—48 patients with OSA	No
Platelet aggregation	Bokinsky, 1995 [57]	Non-randomized study—6 patients with OSA	Yes, no effects
	Sanner, 2000 [66]	Non-randomized study—17 patients with OSA	Yes, decreased after 6 months
	Zhang, 2003 [46]	Non-randomized study—41 patients with OSA	Yes, decreased after 30 days
	Kontos, 2020 [68]	Non-randomized study—30 children with SDB	No
ADP	Alkhiary, 2017 [69]	Case-control study—64 patients with OSA	No
	Oga, 2009 [67]	Non-randomized study—58 patients with OSA	Yes, decreased in platelet aggregation
VWF	El Solh, 2008 [76]	Non-randomized study—35 patients with OSA	No
	Phillips, 2012 [77]	Randomized, placebo-controlled crossover study—28 patients	Yes, decreased in VWF level after 2 months
	Von Kanel, 2007 [75]	Cross-sectional study—135 patients	No
	Von Kanel, 2016 [49]	Longitudinal study—329 patients with OSA	No

ADP: adenosine diphosphate, PAF: platelet-activating factor, sCD40L: soluble CD40 ligand, vWF: von Willebrand factor.
